# RoBétArmé Project: Human-robot collaborative construction system for shotcrete digitization and automation through advanced perception, cognition, mobility and additive manufacturing skills

**DOI:** 10.12688/openreseurope.16601.1

**Published:** 2024-01-03

**Authors:** Ioannis Kostavelis, Lazaros Nalpantidis, Renaud Detry, Herman Bruyninckx, Aude Billard, Schlette Christian, Marc Bosch, Konstantinos Andronikidis, Henrik Lund-Nielsen, Pedram Yosefipor, Usman Wajid, Rahul Tomar, Fernando LLano Martínez, Federica Fugaroli, Despoina Papargyriou, Nikolay Mehandjiev, Gash Bhullar, Estefânia Gonçalves, Jonas Bentzen, Mads Essenbæk, Christian Cremona, Mary Wong, Marcos Sanchez, Dimitrios Giakoumis, Dimitrios Tzovaras

**Affiliations:** 1 Information Technologies Institute, Centre for Research and Technology Hellas, Thessaloniki, Greece, 57001, Greece; 2 Department of Electrical and Photonics Engineering, Technical University of Denmark, Copenhagen, Denmark; 3 Department of Mechanical Engineering, Katholieke Universiteit Leuven, Leuven, Flanders, Belgium; 4 Ecole Polytechnique Federale de Lausanne, Lausanne, Vaud, Switzerland; 5 Faculty of Engineering, University of Southern Denmark, Copenhagen, Denmark; 6 Robotnik Automation S.L., Valencia, Spain; 7 ANiMA Technical Commercial SA, Athens, Greece; 8 COBOD International A/S, København, Denmark; 9 Information Catalyst Sl., Alicante, Spain; 10 DigitalTwin Technology GmbH, Cologne, Germany; 11 Ingenieros Asesores De Construccion SL, Asturias, Spain; 12 UNI - Ente Italiano Di Normazione, Milano, Italy; 13 TITAN Cement Company SA, Athens, Greece; 14 Digital Systems 4.0, Plovdiv, Bulgaria; 15 European Factory Foundation, VIENNA, Austria; 16 MORE - Laboratorio Colaborative Motanhas De Investigacao Associacao, Bragança, Portugal; 17 Christiansen and Essenbæk A/S, Copenhagen, Denmark; 18 Bouygues Construction SA, Versailles, France; 19 Arup Group Limited, Dublin, Ireland

**Keywords:** shotcrete application, construction collaborative robot, autonomous maintenance and inspection, additive manufacturing, Digital Twin, CIM and BIM, IoT, decision-making

## Abstract

The importance of construction automation has grown worldwide, aiming to deliver new machineries for the automation of roads, tunnels, bridges, buildings and earth-work construction. This need is mainly driven by (i) the shortage and rising costs of skilled workers, (ii) the tremendous increased needs for new infrastructures to serve the daily activities and (iii) the immense demand for maintenance of ageing infrastructure. Shotcrete (sprayed concrete) is increasingly becoming popular technology among contractors and builders, as its application is extremely economical and flexible as the growth in construction repairs in developed countries demand excessive automation of concrete placement. Even if shotcrete technology is heavily mechanized, the actual application is still performed manually at a large extend. RoBétArméEuropean project targets the Construction 4.0 transformation of the construction with shotcrete with the adoption of breakthrough technologies such as sensors, augmented reality systems, high-performance computing, additive manufacturing, advanced materials, autonomous robots and simulation systems, technologies that have already been studied and applied so far in Industry 4.0. The paper at hand showcases the development of a novel robotic system with advanced perception, cognition and digitization capabilities for the automation of all phases of shotcrete application. In particular, the challenges and barriers in shotcrete automation are presented and the RoBétArmésuggested solutions are outlined. We introduce a basic conceptual architecture of the system to be developed and we demonstrate the four application scenarios on which the system is designated to operate.

## Plain language summary

The RoBétArmé European project targets the Construction 4.0 transformation of the construction with shotcrete with the adoption of breakthrough technologies such as sensors, augmented reality systems, high-performance computing, additive manufacturing, advanced materials, autonomous robots and simulation systems, technologies that have already been studied and applied so far in Industry 4.0. This paper showcases a case study on which novel robotic systems will be developed for the automation of shotecrete application. The outcomes of this research can be widely used in other application technologies related to the construction domain.

## Introduction

According to the World Economic Forum, the construction industry currently accounts for about 6% of the world gross domestic product (GDP) ^1^ and is expected to reach around 14.7% in 2030 ^2^, which means that the construction sector plays a key role in any country’s economy. At European level, construction is a strategically important sector for the economy involving a wide range of stakeholders and companies, providing 18 million jobs ^3^. There is an immense need of construction automation aiming to introduce new technological solutions for the construction automation of diverse infrastructure such as roadworks, tunnels, *etc.* This need is mainly driven by (i) the shortage and rising costs of skilled workers, (ii) the tremendous increased needs for new infrastructures to serve the daily activities and (iii) the immense demand for maintenance of ageing infrastructure ^4^.

To achieve the desired level of automation, analogous to the Industry 4.0, breakthrough technologies such autonomous robots, vision sensors, augmented reality systems, high-performance computing, additive manufacturing, advanced materials, and simulation systems, have been adapted for construction applications and this transformation has been called Construction 4.0 ^5^. One might reasonably think that Construction 4.0 could bring equivalent benefits to construction domain, by automating traditionally manual, laborious, repetitive, and unhealthy for human workers construction activities. However, construction has been slow to adopt new technologies and has not undergone a major disruptive transformation ^6^. This slow adoption pace is mainly due to the great barriers imposed by the custom, diverse and laborious construction and repair activities required for the construction and/or maintenance of civil infrastructure ^7^. These barriers include: (a) the incompatibility of the new technologies with existing construction practices that forces workers to prefer the former and proven solution instead of innovative technologies; (b) the fragmented nature of the construction industry that inhibits the implementation of new technologies; (c) the high sophistication of digital technologies that prohibits their adoption from less technological familiar construction workers; (d) the fit-to-purpose automation solutions that address unique construction activities bring high investment costs and hinders their accessibility from small and medium enterprises ^8^.

**Figure 1.**
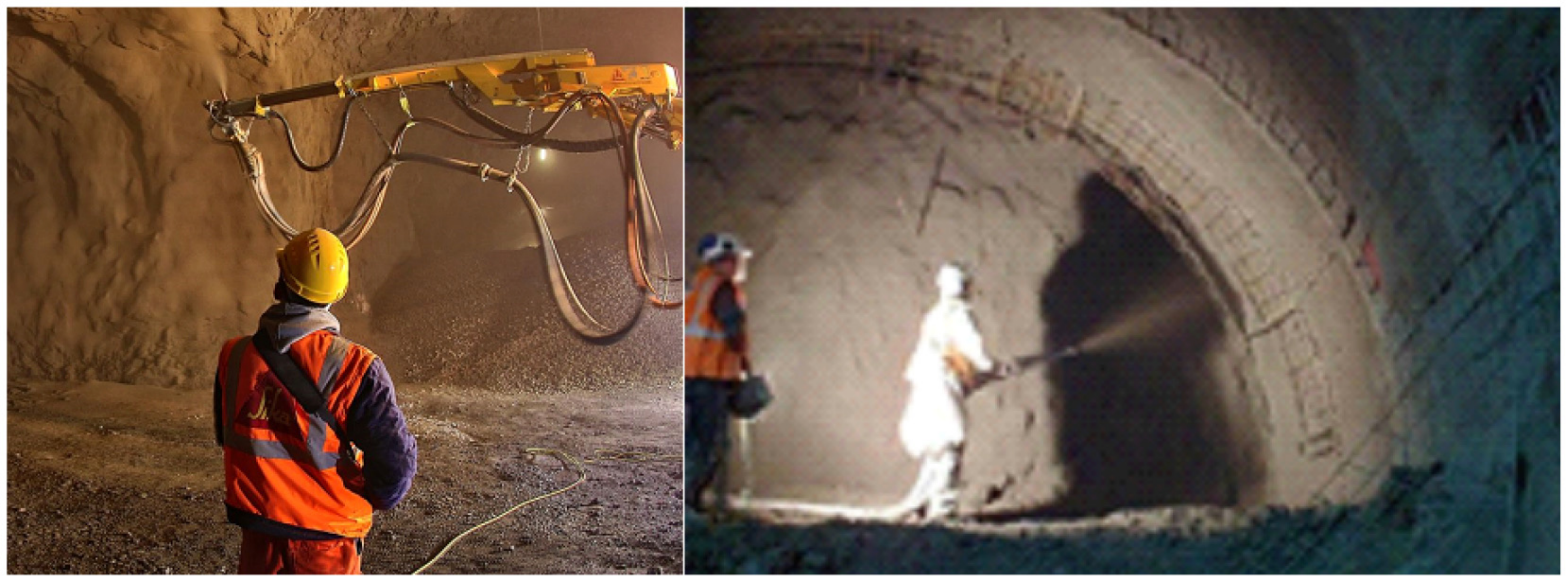
Example of current shotcrete activities. (Images taken by authors during relevant construction activities).

According to construction market needs, the increase in mining activities around the world ^9^, the increase in tunnel construction due to rapid urbanization in emerging economies ^10^, and the growth in construction repairs in developed countries demand excessive automation of concrete placement ^11^. Shotcrete (sprayed concrete) is increasingly becoming popular among contractors and builders, as its application is extremely economical and flexible ^12^. The market size of shotcrete/sprayed concrete is projected to reach USD 8.30 Billion by 2021, at a compound annual growth rate (CAGR) of 8.0% and Europe is the largest market for shotcrete, which accounted for the maximum share of the overall market, in terms of value ^13^. Rapid developments in technology and raw materials, economic and technical efficiency of shotcrete, increase in underground constructions activities such as mining and tunneling are the key drivers for the growing shotcrete demand, while the shotcrete industry is seeing intense research activities to automate existing and create new equipment ( Figure 1), 12

### Construction with shotcrete - the need for automation

Shotcrete ( *i.e.* concrete spray casting), refers to the pneumatically applied mortar or concrete on a surface and constitutes the most widely used technology in underground mining, tunnel construction and surface rehabilitation of infrastructures ^14^. Shotcrete is a typical example in the construction domain, where even though a highly mechanized procedure (nozzles, pumping machines, mortar-mixers, telescopic reach tractors, *etc.*), the quality of sprayed concrete application heavily depends on the operators’ skills. Equipment manufacturers struggle to develop shotcrete automation solutions to minimize variations among operators to increase productivity and safety. Yet, the current technology significantly varies and depending on the application, the shotcrete can be manual ^14^, ( *e.g.* handle the nozzle in narrow culverts, buildings) or semi-automatic ( *e.g.* teleoperate a shotcrete boom in tunneling, mining) ^15^. Specific factors emerge from the automation of shotcrete application ^16^:

1. The thickness of shotcrete applied depends on the skills of nozzlemen, considering that the generated dust blocks the operator’s line of sight who cannot directly assess the surface condition and the control of the nozzle is applied empirically.
2. The compliance of the sprayed concrete thickness with the design requirements [( *i.e.* computer aided design models (CAD)] is not known until after a survey is completed. The survey is manual and relies on expertise of workers to identify areas of over spray or under spray.
3. (To control the quality of application, nozzlemen use depth pins or string lines as guidance to allow them to (visually) gauge the approximate depth of concrete placement, which is a time consuming and labor-intensive task.
4. To reduce the amount of under- spray sections and prevent rework, nozzlemen place more shotcrete than required, wasting significant amounts of material and water.
5. The rebound phenomenon during shotcrete produces large amount of dust containing quick-setting agents, which inhaled by workers may cause great harm to the health ( *e.g.* pneumoconiosis, cancer, *etc.*).

### Technological challenges in shotcrete automation

Up to now, only a few highly automated solutions have been applied in the construction domain and such solutions only scratched the surface of shotcrete automation ^4^. The challenge is to deliver a highly digitized and robotic-enabled solution robust and versatile enough to perform autonomous shotcrete in diverse construction environments *e.g.* tunnels, buildings, bridges, retaining walls, *etc.* However, important technological aspects need to be addressed. The environment continuously evolve during the construction activities, rendering the 3D registration with topographic drawings challenging ^7^. The absence of building/construction information models (BIM/CIM) in ageing infrastructure to be maintained/repaired requires on-the-fly modeling of the surface to be spray-casted with a plethora of manufacturing parameters needing optimization ( *e.g.* mortar viscosity, need for additives *etc.*) ^17^. Small and sparse defects in rebar/metallic reinforcement should be fixed prior to shotcrete applications, which requires high precision vision methods to guide the welding machinery. The variation of construction sites introduces diverse illumination conditions (from completely dark sites in *e.g.* bridges post-tensioned boxes to direct sunlight exposure in retaining walls construction sites) which require different sensors types ^17^. The produced dust during the shotcrete creates a turbid environment that renders the *in-situ* perception feedback difficult and the closed-loop comparison with the CAD reference model cannot be solved with trivial computer vision. Real-time control of the (pneumatic/hydraulic) valve should be performed in parallel with dexterous manipulation to ensure accurate shotcrete placement ^18^. The harsh and uneven terrain in construction sites provokes the vehicle’s navigation where increased stability is required to compensate puddles and rocks that cancel the wheel odometry (necessary in GPS-denied sites). The surface finishing requires complex manipulation motions to achieve the required quality and surface roughness estimation with vision methods is demanding since real-time inference is required to close the loop with the scrapping system.

## The RoBétArmeé project in a nutshell

### Conceptual description

**Figure 2.**
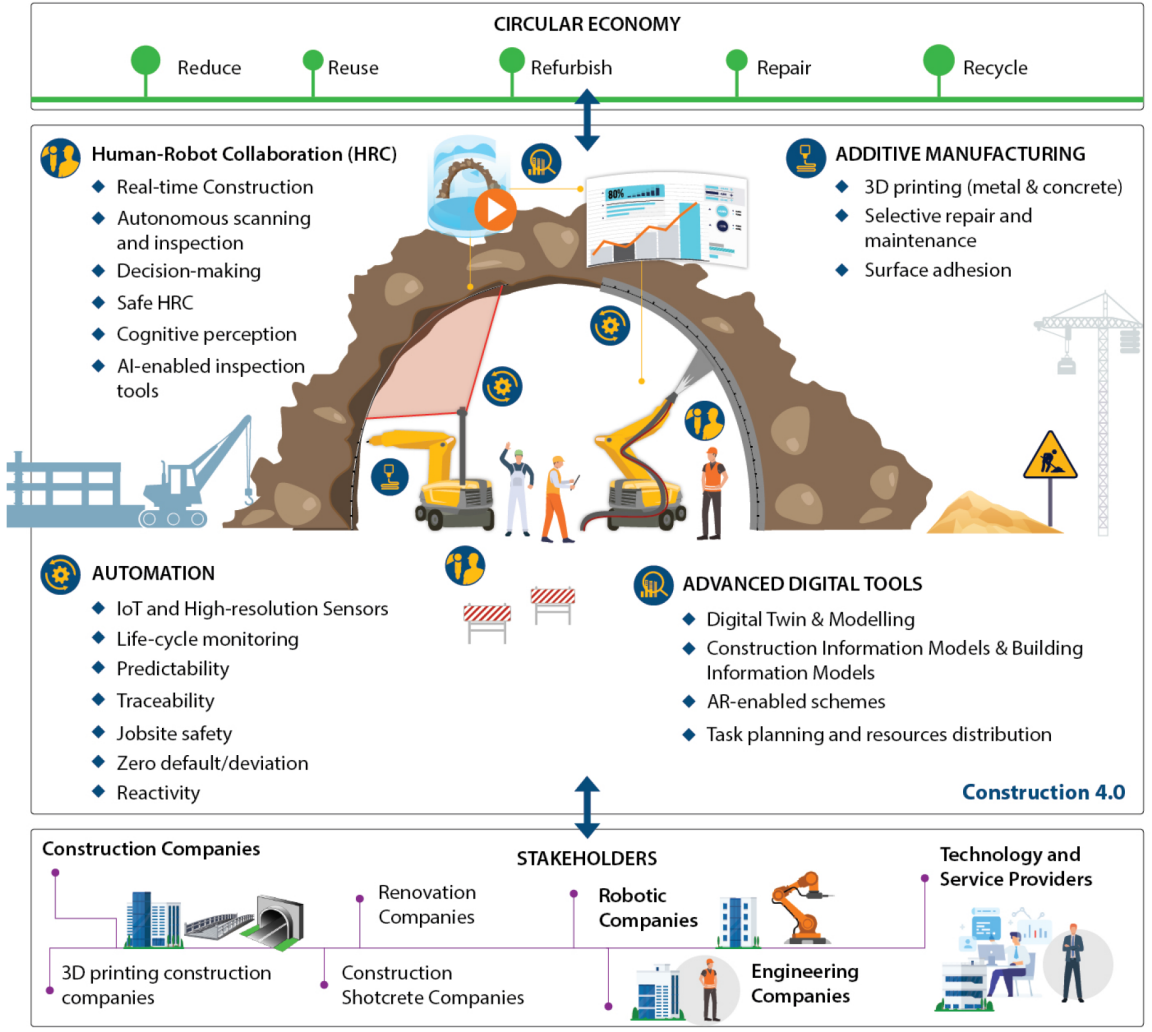
RoBétArmé responses in Construction 4.0 application areas.

RoBétArmé ^19^ aims towards a step change in the Construction 4.0 ( Figure 2) by automating particularly laborious construction tasks in all phases of shotcrete application. To this end, RoBétArmé will deliver collaborative construction mobile manipulators, consisting of an (i) inspection-reconnaissance mobile manipulator (IRR) to address fast, high precision modeling and rebar reinforcement through metal additive manufacturing in the preparatory phase and (ii) a shotcrete and finishing mobile manipulator (SFR) to address autonomous shotcrete application and surface finishing during the construction and finishing phase, respectively. Specifically, in the preparatory phase, the multitasking IRR robot will leverage the latest geotechnical monitoring, data form high-resolution vision sensors and high-speed computing technologies to automate the modeling and fast reconstruction of the surface to be shotcreted. IRR will also facilitate rebar reinforcement through metal additive manufacturing (AM) capitalizing on its precise repair skills. In the construction phase, the SFR robot will apply dextrous concrete placement through the visual guidance of the advanced perception system. The prior and *in-situ* digitalization methods will provide real-time feedback to the construction robot improving the efficiency and quality of concrete spay casting, while reducing waste in construction. This modular robot endorsed with dexterous end-effectors (shotcrete nozzle and scrapper) will automate the third construction phase, by applying delicate and human-like surface finishing skills. In all phases, RoBétArmé will provide a digital twin and advanced simulation tools tailored to the BIM/CIM models for the fast and greener implementation of the automated construction activities.

### Technological objectives and contributions

The RoBétArmé project will contribute in the transformation of Construction 4.0 thought the development of a series of technological solutions adopted from Industry 4.0. Its main objective is to deliver novel cognitive robot platforms that address all the three phases of shotcrete application for autonomous construction, maintenance and monitoring activities of infrastructures. In particular the IRR robot will be endowed with cognitive perception capabilities that fuse multimodal sensors for the high precision modeling of the construction site. This robot will be also endorsed with a metal AM manipulator to perform reinforcement of metallic rebar, cleaning of rusted rebar, placing markers for the shotcrete monitoring *etc.* The SFR robot will perform wet and dry shotcrete through concrete spay-casting, relied on visual guided robotic manipulation and human-robot shared control for workers’ skills transferring. In addition, a real-time and anticipatory digital twin and simulation environment will allow the lifelong monitoring of the constructed/repaired site with smart, low-energy IoT sensors. On top of the system a multimodal human-robot collaboration (HRC) suite will enable high quality construction. Next, the main technological tools that will operate on the robots are summarized as follows:

1. *To deliver an advanced high-precision and real-time infrastructure perception system through artificial intelligence (AI) -enabled technologies and multimodal sensor fusion.*
  An AI-based cognitive perception system integrated on the IRR and SFR robots will offer fast processing of data stemming from diverse modalities and precise digitalization and modeling of the construction activities. In preparatory phase a georeferenced high-precision geometric and semantic digital model will be created exploiting onboard high resolution sensors ( *e.g.* laser imaging, detection and ranging (LiDAR) and laser scanners) accompanied with novel and beyond state-of-art vision-based simultaneous localization and mapping (SLAM) approaches. Closed loop, high-precision visual servoing will allow the rebar reinforcement with metal additive manufacturing and low energy consumption Internet of things (IoT) sensors (humidity, vibrations, temperature, corrosion *etc.*) placed on the constructed surface will enable future monitoring and diagnostics of the construction degradation. In the shotcrete phase, the cognitive perception system will provide robust representations of the harsh conditions of the construction site, compensating the dust effect, during concrete spray casting, through real-time visual wall thickness sensing and semantic material/environmental understanding. In the finishing phase, an *in-situ*, vision-based quality control layer will continuous monitor the surface quality and roughness, through AI-based methods, to ensure high quality result (WP5).
2. *To deliver cognitive and adaptable human-robot collaboration control schemes for dexterous execution of construction and maintenance tasks.*
  The IRR robot will be equipped with specifically designed and integrated metal-additive manufacturing nozzle to achieve direct metal printing and/or rebar cleaning from rust. The SFR robot will perform the shotcrete and the surface finishing tasks by removing the over-spayed material *i.e.* scrapping. To this end, the SFR robot will be equipped with modular shotcrete nozzles capable of performing either wet or dry shotcrete tasks. Actuation of heavy-duty mechatronics systems will be applied through cognitive action planning, motion and control. A) Trajectory planning and precise closed-loop control methods for metal additive manufacturing and shotcrete application will be delivered. B) Safe control and robot reaction strategies that account for human presence will be realized. C) A high fidelity coupled sensing and actuation control will enable physical and dexterous concrete placement through real-time visual wall thickness monitoring, d) shared-control methods that enable physical human-robot collaboration for the surface finishing and scrapping will expedite the refining step of shotcrete
3. *To release advanced modeling tools tailored to the building and construction information models for the fast and greener implementation of automated construction activities.*
  RoBétArmé foresees the realization of collaborative construction robots as an advanced tool for the human workers. The human presence modeling will be performed by fusing information from input provided through intuitive human machine interaction interfaces ( *e.g.* login information, stored interaction preferences, user experience), personalized ergonomic parameters and vision-based human detection. Next, complex environment modeling is foreseen by combining information from the initial robot scanning/mapping, the existing CAD data along with the CIM and/or BIM, to formulate an up-to-date model of the construction environment. On a lower modeling level, a detailed model of the construction area will be designed by fusing visual and non-visual means so as to outline the 3D structure required for the construction 3D printing process (metal additive manufacturing and shotcrete). Last, the concrete recipe will be defined by incorporating details such as the binder material for adhesion during shotcrete, the chemical composition of the concrete to be spray-casted targeting to lower the CO _2_ emissions in the entire shotcrete application.
4. *To introduce a cognitive digital twin and simulation environments for construction monitoring, diagnostics and orchestration activities.*
  The RoBétArmé will deliver pioneering technologies for construction robots especially in the limited, so far, explored domain of mobile 3D printing (metal additive manufacturing and shotcrete) for construction and repair. In this respect, an advanced cognitive simulation tool will be developed, within this project, to serve both as a construction digital twin for real time construction monitoring as well as for the micro-assessment and simulation of the models to be constructed. Model slicing activities and layer decomposition for nominal shotcrete and metal 3D printing nozzle trajectory will be part of this simulation, while also simulations with finite element analysis will assess different chemical compositions of materials in terms of their durability, and stamina. The system will infer a high-level construction plan in order to coordinate the ongoing procedures. Tasks orchestration and adaptation will be also seamlessly integrated into the Digital Twin ecosystem where the operator will have the capability to examine multiple inspections and construction scenarios before the actual robots’ deployment.
5. *To deliver an adaptable and safe construction planning and orchestration that respects construction environment, physical and process-oriented constraints.*
  The RoBétArmé robots will be safe-by-design through embedded safety planning manipulation mechanisms: a) A structured task planning and orchestration tool will sequentially address the subordinate actions for the execution of the robots perception and construction goals, by fusing robot manipulation skills coupled with ubiquitous inherent safety controllers. b) A novel stochastic manipulation planning scheme that i) respects and continuously avoids human presence, ii) considers the environment and material constraints ( *e.g.* concrete viscosity, time to stabilize, adhesive materials, air and water pressure valve control) during the deposition of different layers, iii) plans the direction of nozzles for optimal construction 3D printing (metal additive manufacturing and shotcrete) and iii) supports human-aware robot navigation, will be developed. c) An AR-based planning scheme for safe collaboration between nozzlemen and system will be realized to increase situation awareness
6. *To demonstrate and evaluate the functional prototype of robotic platforms in four real diverse construction sites.*
  The RoBétArmé robots and digitilization tools will be demonstrated and validated through real diverse construction sites across different countries in Europe covering shotcreting for (1) ground support (retaining) walls, (2) tunnels and/or culverts, (3) beams & piles of buildings, and (4) the posttensioned boxes of bridges, provided by the project end-users.

## Related research efforts in construction domain

The main technological breakthrough so far in construction domain is the adoption of BIM, on which also standards have been created ^20^.

Since its debut, governments around the world have rapidly adapted it for a great variety of construction activities ^17^. In BIM construction workflow the design and authoring of the architectural and tunnel designs are done in 3D CAD software, such as Revit and Civil3D, where a full 3D model of the completed section is created. This is followed by the virtual design and construction (VDC) process where a complete construction simulation is run using the CAD models.This helps validate the both the construction process and the schedule. Clash detection is also accomplished with the design model to detect any potential design conflicts before the construction plan is approved. The introduction of BIM dominated the rest of the technological advancements in the construction domain, and since then, slightly any generic and standardized information and communication technologies have been established in such grade or their are adopted with a slow pace ^21^.

### Relevant European projects

In order to expedite the Construction 4.0 transformation in Europe, the European Union in the context Horizon Europe work program, in the wider spectrum of twin green and digital transformation initiated a specific topic seeking breakthrough technologies that support technological sovereignty in construction. In the context of this work program apart from RoBétArmé, two additional European projects have been funded aiming to develop digitalization tools for the construction domain.

The project HumanTech ^22^ aims to develop innovative, human-centred technologies that go beyond the current state of the art by contributing to the digitalization of the construction industry, making it safer and more productive, encouraging a new generation of highly skilled professionals, and accelerating the transition to green construction. The project involved robotic devices equipped with vision and intelligence that will be able to navigate autonomously and safely in highly unstructured environments, collaborate with humans and dynamically update a semantic digital twin of the construction site in which they are. Visual information capture will be extended to multispectral images, which will allow detecting the material composition of constructions, besides their geometric characteristics. For workers foresees the utilization of smart unobtrusive protection and support equipment, including exoskeletons activated by body sensors for posture and strain to wearable cameras and extended reality (XR) glasses that provide real-time workers’ location and guidance for them to operate efficiently and accurately. Also, the project develops a novel breed of Dynamic Semantic Digital Twin that they will allow simulating the current state of a construction site in detail, at the geometric and semantic level, based on an extended Building Information Modeling (BIM) formulation that contains all relevant structural and semantic dimensions (BIMxD). These will act as a common reference for all human workers, engineers and autonomous machines.

The project Beeyonders ^8^ has in its core ambition to address the challenges (mentioned in Sect. by producing, commercializing and integrating beyond the state-of-the art solutions adopted from Industry 4.0, into real construction scenarios. In order to achieve this, the project will make extensive use of artificial intelligence (AI), automation, and digitisation. These new technologies will both support the independence of the European construction sector from foreign technology imports and reduce its environmental impact, ultimately supporting the EU in reaching climate neutrality. In more details, the project seeks to to improve improving efficiency, safety, and quality in the construction sector while reducing the environmental impact of building sites. In order to achieve than, Beeyonders will integrate autonomous vehicles and human robot collaboration, additive manufacturing, diagnostics and monitoring and autonomous maintenance to successfully operate in the construciton sites. It will demonstration the impact of the usage of these new pioneering technologies on the efficiency of resources (raw materials, water *etc.*) and in the reduction of waste and embodied CO2 emissions. Moreover, Beeyonders contributes also in the safety of the utilization of such new technologies in a construction environment, in cooperation with workers. Also the projects through the envisioned technologies aims to increase in the wellbeing of the construction workforce involved in mundain activities.

It is apparent that the technologies introduced in the construction sector by these two European projects are complementary to the technologies that RoBétArmé is going to develop and all of them are targeting to the increase of sovereignty in the construction domain. Core research is applied in all cases for the transformation of the technologies used in Industry 4.0 and health domain into viable solutions to the challenging environments of construction sites.

### Existing technological solutions and RoBétArmé responses

***Mobile robot manipulators in construction.*** Robots designed for interior building finishing, brick laying masonry and the development of robot-based prefabrication of facade and wall elements ^23^, have been delivered. However, these concepts have not reached the market, nor became reference applications for construction automation, since the proposed technologies are not planned to be multipurpose and do not safety and standardization.

RoBétArmé will deliver the IRR and SFR mobile manipulators to automate significant aspects of shotcrete application in modular manner. IRR will be a modular multipurpose robot that performs high digitalization of construction site and also repairs/constructs the metallic reinforcement net with mobile metal 3D printing. The SFR multipurpose modular platform will perform the concrete spray casting and will be reused (with some tool-changing) for the surface finishing.

***Concrete additive manufacturing.*** The most widespread technology for large-scale concrete components is the layer-based extrusion technique, where concrete layers are deposited upon each other creating that way the desired product ^24^, providing design flexibility ^25^. A drawback is that the concrete is printed in vertical axes that limits construction abilities, and the slow printing speed hiders the efficiency in construction. In contrast, the shotcrete 3D printing technique sprays a dispersed stream of concrete with pressure to build a 3D structure proving a series of advantages and possibilities ^26^, such as multi-orientation and high speed. Yet, the projected concrete is open loop and the result structure is manually compared with the nominal CAD files ^27^. Recently, shotcreting witnessed some technological advancements where automation applied to the remote teleoperation of the shotcrete nozzles.

The core innovation of RoBétArmé is the delivery of the first construction mobile manipulator that will perform shotcrete application with real time robotic perception input. This will be achieved by firstly creating –at the construction site- the digital model of the area to be scanned and, secondly, after parsing this model, the autonomous application of shotcrete with robotic manipulations will cover the reconstructed area. On-board real time perception system ^28^ will increase monitoring efficiency and will allow for accurate guidance and placement of the concrete closing the loop among the desired CAD model to be built and the actual shotcrete.

***Metal additive manufacturing.*** To enhance tensile strength and to be able to carry higher loads in shotcreting, research has been done with various fibers or metal wires ^29^. Metal 3D printing has seen publicity ^30^ and various technologies are currently in use *e.g.* the Powder-bed Fusion main category has sub categories such as Direct metal laser sintering (DMLS), selective lase melting (SLM), electron beam melting (EBM) and direct metal printing (DMP). Albeit the fact that most of these technologies, require a highly controlled, industrial environment the direct energy deposition (DED) technology, frequently used for repairs of metal parts.

DED metal 3D printing technology will be integrated on IRR robot to perform *in-situ* reinforcement of the rebar prior to shotcrete application. Coupled with the perception system that will infer areas where the metallic net required reinforcement, the metal structure will be built layer by layer, during the preparatory phase. This way we can have a truly solid metal skeleton in concrete structure, avoiding on-site manual welding applications ^30^.

### Digital Twin and modelling (BIM/CIM)

In generating digital twins of bridges and buildings, after reconstructing the model, the designers enrich it by space detection which in the field of computer vision, be implemented in many different approaches, such as camera-based reconstruction, 3D photogrammetry, deep visual and semantic SLAM, and so forth. Digital twin models cannot be compiled by the staff of building or infrastructure owners, for two reasons: a) highly specialized knowledge is needed for compiling digital twins, and b) the effort for compiling a digital twin is concentrated at the start of their life (whether from existing infrastructure or at the handover from a project built with BIM) ^31^.

In RoBétArmé specific steps to enhance the advancement of digital twin within the project will be followed: (a) Real-time synchronization and monitoring, knowing what IoT standards are best-suited for these operations will enhance the acceptance of digital twin and make it easier for widespread adoption. (b) Integration of construction specific semantics and model encoding in a universal format to be accessible by robots. AR-tools *etc*.

### High resolution 3D reconstruction and multimodal sensor fusion

3D reconstruction finds applications in civil engineering structures ^32^. Currently, SoA methods make use of deep neural networks to create 3D models from multiple images ^33^. In most practical cases, SoA methods fuse multiple modalities, most commonly vision (capturing context and semantic information) and LIDAR data, to get high accuracy ^34^. As multiple sensor data streams need to be fused attributing varying significance to the various inputs, situational aware solutions exploiting Recurrent Neural Networks, or Transformer models ^35^ are often used.

High resolution and accurate 3D reconstructed models will be achieved through multimodal sensing.We will exploit multitask learning to share some of the neural network layers among tasks that can support one another, such as *e.g.* keypoint detection, edge detection, semantic segmentation, *etc.* While, research indicates that multitask learning allows the networks to learn better features during common training, this approach requires a deeper knowledge of how these networks learn, and the loss functions become exponentially more complicated in order to balance the different tasks during backpropagation ^36^. RoBétArmé will use for the first time multitask learning to assist multimodal 3D reconstruction, achieving 3D modeling accuracy.

### Cognitive robot manipulation and control

Robotized maintenance tasks are restricted to inspection and monitoring that rely on dexterous navigation and perception but require little or no manipulation, ^37^. Maintenance tasks in space ^38^ require series of manipulation but current techniques rely largely in traditional planning, and do not require fast re-planning in the fact of disturbances and obstacles, given the slow tempo of motion in space. Manipulation, required for shotcrete, were until recently mostly teleoperated ^15^. Recent works offer automated solutions ^39^, but automation pertains to positioning properly the robot’s base, with no control of the arm, nor of the spraying amount and resulting interaction forces.

RoBétArmé manipulation and control competences builds state of art controllers for on-line trajectory planning and force adaptation with theoretical guarantees for convergence, stability and passivity ^40^. By deploying these techniques in shotcrete, it will show-case the applicability of these controllers in a real environment, and aims to bring these technologies to TRL5. Abilities for self-monitor task completion for surface finishing will be delivered and to autonomously re-planning of arm and base trajectory for proper task completion while navigating to the site will be addressed ^41^. It will use trajectory-based control for shotcreting and force control for scraping.

### Vision systems in harsh environments

Turbidity impedes vision sensors that rely on visible or near-infrared (IR) wavelengths ^42^. To allow robots to work in turbid conditions, the first line of recourse is to use longer wavelengths such as radar or thermal imaging, but their lower spatial resolution is unfortunately often prohibitive. The mitigation of the effects of turbidity with near-visible–light sensors is carried out by first detecting then filtering its effects ^43^. Alleviation is typically conducted through image filtering ^44^ or object filtering ^45^, sensor fusion, and application-specific solutions that exploit specificities of a given task to improve the information content of sensor data ^46^.

RoBétArmé will exploit a variety of sensors in concert and will go beyond the SoA by designing a shotcrete-specific sensor system, configurable to individually address the different environmental situations we expect to face. Each situation will be semantically modeled with cause-and-effect relations of identified sources of disturbances to performance, uncertainty and confusion of the sensor processing.

### Robot cognitive functionalities for autonomous operation

The robots autonomy relies on their capacity to decide on their own actions based on their cognitive functionalities and realize these actions using their robotic planning mechanisms ^47^. In the construction domain, complete situation awareness is not feasible since the environment should be modeled with specific robot sensors that produce noisy measurements. Task planning requires that several types of knowledge is encoded in the planner in a suitable way (finite state machines). These include causal knowledge about the effects of the robot’s actions, and knowledge about the objects in the world, their properties and their relations ^48^. In construction robots, autonomous decision making and task-planning have not been studied, since most of the robotized solutions are teleoperated.

RoBétArmé will transform, modify and adopt the robot decision-making tools developed for Industry 4.0 applications in the construction domain. The high-level decision maker will be a self-explainable and human-understandable network that will assess the current construction state and the agents and will infer the next best action. A low-level active task planner as a self-organized FSM will recall/trigger the set of robot skills that will facilitate the selected construction action.

### 0.0.1 Environment semantic understanding

While in public areas the semantics mostly follow strict rules defined by the guidelines and laws, construction sites have their own rules of layout. The road, construction vehicle parking area, personal vehicle parking area, sediment area, and construction material area ^49^ are not strictly defined but are decided by a multitude of factors ^50^. Understanding the semantics of an environment is essential for human workers and autonomous construction vehicles/robots. In order to understand the semantics of construction sites, it is necessary to observe the site over a longer period and deploy algorithms that account for the environment evolution of dynamic construction sites.

RoBétArmé will deliver semantic mapping methods tailored to dynamic environments such as the construction sites modeling the behavior (spatial and temporal) of all the involved actors. Building in our previous work ^51^, representation of temporal objects persistence in the construction environment will be applied to model static and dynamic variations including the evolution of the construction site through deep neural networks, and the spatial imprint of these representations on the workspace will facilitate the robots’ navigation skills.

### IoT based structural health monitoring

Up to now, effective implementation for IoT used for monitoring regular domestic conditions ^52^ by means of low cost ubiquitous sensing system has been used in the field of construction informatics to address structure health monitoring (SHM). The used sensors are typically superficially installed on home environment to measure temperature, light, humidity, *etc*, through a wireless network. To achieve active SHM sensors should be implanted on the vital parts of the buildings ^53^.

RoBétArmé foresees actual low voltage sensor implantation on the structure to ensure future SHM in construction. The 3D printed concrete pieces created by the RoBétArmé robotics will embed digital sensors (vibrations, bending, humidity, *etc.*) for environmental monitoring inside the constructed area, using ultra-low powered sensors,with open interoperability IoT protocols ensuring integration with a digital twin.

## RoBétArmémethodology

### The automation procedure

**Figure 3.**
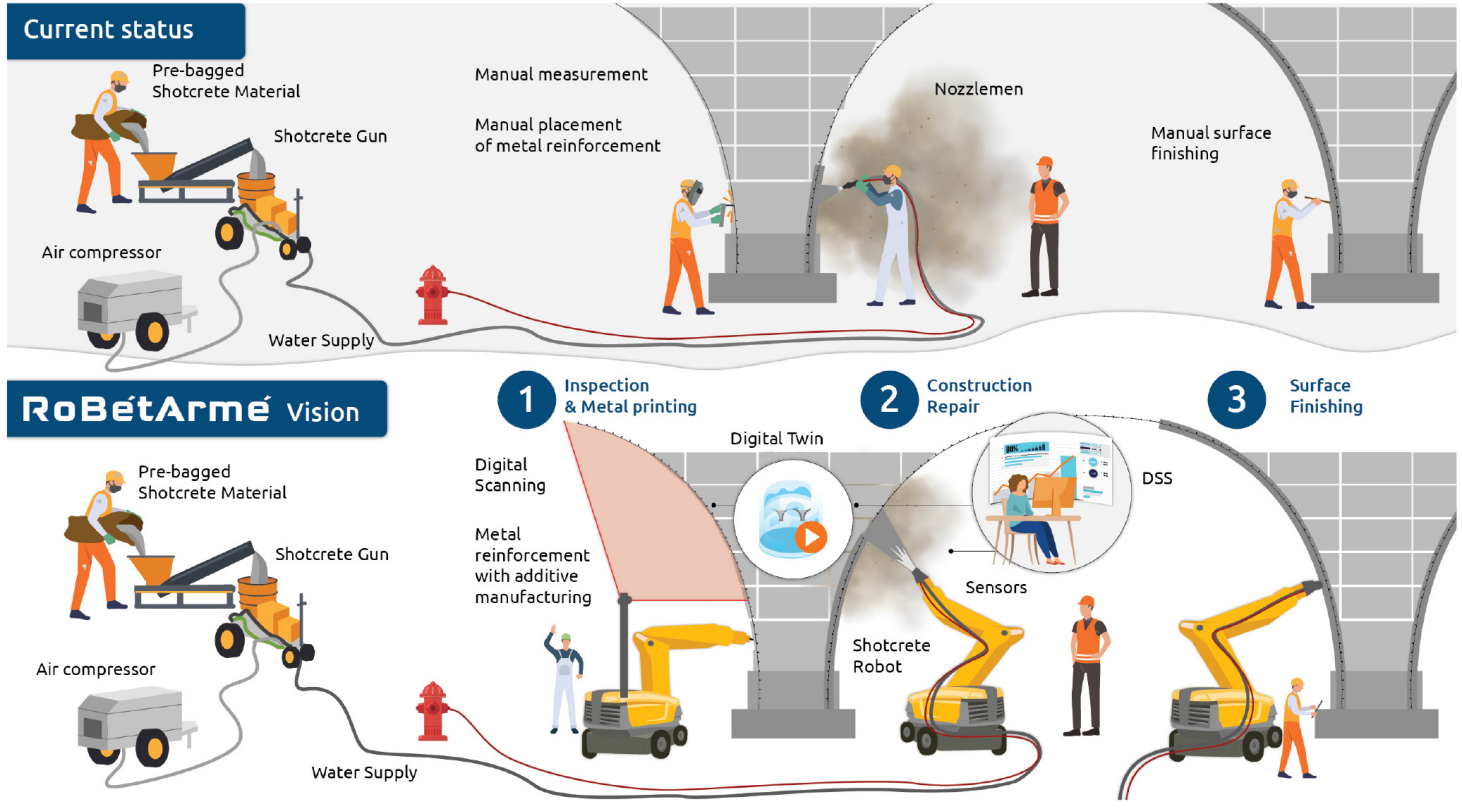
RoBétArmé Overall Vision and Automation Concept.

Shotcrete (wet or dry) is a mortar or high performance concrete conveyed through a hose and fluid projected at high velocity onto a backing surface. Shotcreting has proved to be the most commonly used method for construction of curved surfaces, it is also used in buildings’ façade restoration after fire or earthquakes as well as on bridges, as stated by the experts involved in this project. At present, most construction companies mainly adopt manual shotcrete operation ( Figure.3 up), which is applied into three major phases, (a) the inspection and preparatory, (b) the construction/repair shotcrete and (c) the surface finishing phase, where currently several manual tasks are performed. In (a), workers manually inspect, clean the site, install markers for shotcrete control and repair/install the metallic reinforcement net. In (b), workers control the shotcrete manipulator through the handheld remote controller to perform spraying or even hold the nozzles with their hands and in (c), the workers employ hand-held tools (trowel or mag) to remove the excessive material. Several problems exist in all shotcrete phases. ( Figure 3 up): The manual installation of markers (bolt-tips, string lines *etc.*) is time consuming, inaccurate and hinders survey information. The inspection with naked eyes of metallic net deficiencies (in harsh environment) typically leads to omitted spots. A large amount of dust generated during the concrete spray casting blocks the operator’s line of sight, and the operator is not able to accurately grasp the condition of the surface to be sprayed and the position and posture of the spraying nozzle, resulting in low spray quality operations. The environmental conditions in the shotcreted areas is complicated and unstable, and there is a risk of rock burst and rockfall, threatening the safety of the workers. The rebound phenomenon during shotcrete produces excessive dust, which contains a large amount of quick-setting agent which inhaled by workers causes great harm to the body. The operator controls multiple handles to manipulate multiple joints of the manipulator, which requires the operator to have excellent skills, yet at present there is a shortage of skilled operators.

The RoBétArmé project as described in Figure 3 below, will deliver digitalization and robotics solutions for tackling the above-mentioned challenges in all the three phases of shotcrete application as follows: (A) In the inspection and preparatory phase: an IRR with advanced multimodal vision system and metal 3D printing arm manipulator, will perform (1) the initial precise 3D reconstruction to obtain the actual contour of the surface to be sprayed. By comparing the actual contour and the design contour (CAD from BIM/CIM), the volumetric area of sprayed concrete required can be calculated, avoiding the manual installation of bolt-tips and markers, used as guiders from the nozzlemen workers. (2) Detection and localization of metallic nets deficiencies and metal connection bonds in the scanned area, through the advanced vision system, and (3) *in-situ* repair/manufacture the reinforcement with the metal 3D printing system of the IRR robot, (4) implantation of IoT sensors for future monitoring and diagnostics of the construction degradation. (B) In the construction/repair phase: the SFR, endowed with perception capabilities for harsh environments, along with a collaborative manipulator equipped with shotcrete extrusion nozzle will undertake to perform the actual shotcreting. This will be achieved (1) by interpreting the 3D model, (2) by inferencing a trajectory plan that guides the spraying nozzle to enable the automatic operation of the shotcrete manipulator. (3) Visual feedback will close-the-loop for the required spayed concrete to match the geometry of the CAD model enabling, this way, real-time inspection of the shotcreted surface. (4) Delicate control of shotcrete nozzle and dexterous manipulations will reduce the rebound effect and thus the hazardous emission of dust. (C) In the surface finishing phase: the SFR, with the appropriate tool changing will undertake to refine the quality of surface. This will be done (1) by spraying a thinner layer (or stiffener) to prevent rework after shotcrete and (2) by changing the tools in SFR with specific designed scrappers used to remove of excess shotcrete material, through dexterous and tangential manipulations. (3) Visual-enabled control that monitors the surface roughness and indicate the spots that need treatment, will expedite the finishing procedure (4) HRC through shared-control strategies will enable human intervention for refining surface finishing. All phases of RoBétArmé envisioned solution will be coupled with a Digital Twin platform that interconnects with human-machine interaction (HMI) interfaces and gathers, analyses and visualizes the ongoing process, suggests through cognitive decision making applications the next actions, enable construction simulation and optimization scenarios and offer increased situation awareness during operation phase. These will contribute in the safe deployment of such technologies in the construction environment and will contribute towards the increase of the well-being of the nozzlemen workers.

### Conceptual architecture

**Figure 4.**
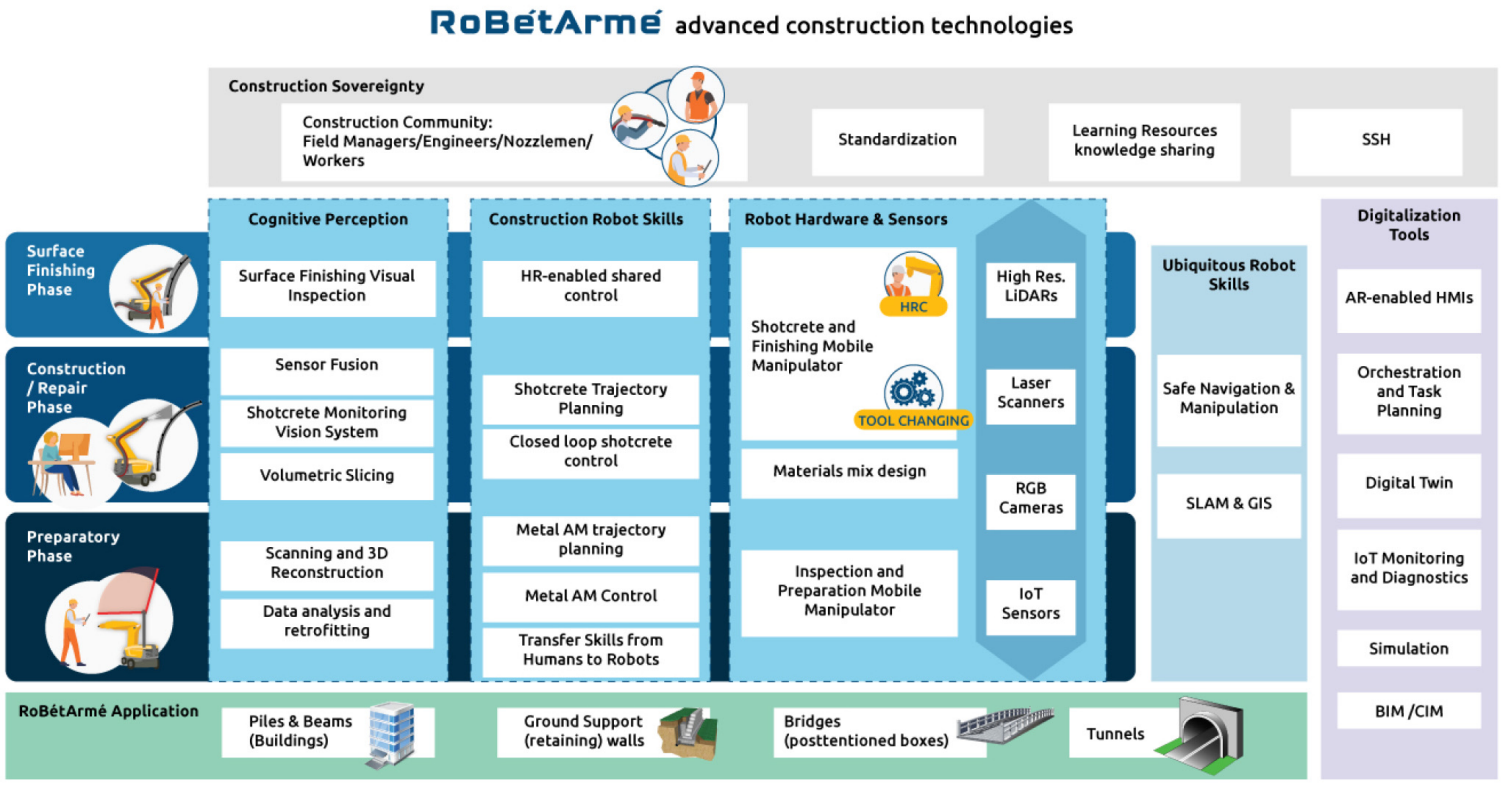
RoBétArmé Conceptual Architecture.

The RoBétArmé architecture follows the three shotcrete application phases exhibiting in each one the advanced technological solutions for the automation of the complete shotcrete application chain. It has been designed in a bottom-up approach, where dedicated software and hardware tools will be developed in each construction phase, while also tools used in all phases and activities will operate during the entire shotcrete operational phase, as illustrated in Figure 4. The RoBétArmé project targets the automation of shotcrete application in four diverse constructions sites, which have been carefully selected based on their significance and frequency in the construction domain, imposing specific challenges to be resolved. This includes i) construction of ground support walls, ii) repair of piles/beams, iii) repair of bridges posttensioned boxes, and iv) construction of culverts or tunnels.

### Inspection and preparatory phase

**Robot hardware and sensors:** The IRR mobile manipulator will consist of multimodal sensors ( *e.g.* LiDARs, RGB and Laser Scanners) and an arm manipulator integrated with a metal extrusion nozzle. It will perform the initial 3D reconstruction of the area to be shotcreted as well as will reinforce the metallic-net/ metallic rebar with Direct Metal Printing to repair existing reinforcement or to increase the adhesion of the surface. Low energy consumption IoT senros will be implanted into the constructed surface to monitor the future degradation of site.

**Cognitive perception:** A scanning and 3D reconstruction module, using the IRR HW, will provide a high resolution model of the operational phase. Precise vision-based methods accompanied with AI-enabled methods ( *e.g.* deep convolutional neural networks) will accommodate the initial modeling exploiting also georeferenced SLAM approaches, enabling the connection of *in-situ* measurements with a complete digital model. The reconstructed surface will be represented as high-resolution meshes, and will be integrated with semantic inferences that can be conceived by the humans. Reinforcement defects and inefficiencies will be also detected and represented into the model through multi-tasking learning-based object detection. Data collection, aggregation and analysis will take place in this phase and IoT sensor information will be passed through the integrated Digital Twin platform to the rest construction phases.

**Construction robot skills:** Firstly, a control and manipulation scheme will be delivered in order to transfer the skills from the nozzlemen workers to the robots. This will be applied in an offline manner during the development step and will realize a library of basic joint motions applied during activities that take place in preparatory phase. The learned construction skills, modeled with vision system, will be transferred to the robot manipulator. Next, and during the operational phase, arm trajectory planning and dynamic system control will interpret the learned skills to perform metal 3D printing procedures, based on existing CAD files generated. The method will allow also shaping the impedance exploiting reinforcement-learning approaches. In this phase, human-centric navigation methods will be developed capable to maneuver with safety the heavy-duty mobile manipulator in the construction site capitalizing on real-time kinematic (RTK) differential GPS-where possible- fused also with vision-based localization techniques.

### Construction/Repair phase

**Robot hardware and sensors:** The SFR robot will be endorsed with advanced vision sensors such as high precision directional LiDARs, RGB stereo sensors and laser scanners. A heavy-duty robot ( *e.g.* Stäubli TX2-160 or a COBOD manipulator) will be utilized integrated with a controllable shotcrete nozzle capable of performing (wet/dry) concrete spray casting, exploiting simple tool changing mechatronics approaches to address situations where hybrid solution is required. A hose management system will be applied on the mobile platform to hold the hoses full with concrete and release the drag and excessive forces ( *e.g.* nozzle kick due to start and stop). A dedicated materials mix design will be studied where the concrete along with reinforcement additives will be mixed taking into consideration all the environmental and the chemical composition attributes of the extruded material, integrating existing mechatronics solution in the shotcrete such as pumps; air compression valves *etc.*

**Cognitive perception:** A volumetric slicing approach will be delivered in order to compare the digital model with the existing georeferenced BIM and CIM and estimate with accuracy the surface to be shotcreted, while extracting an initial construction trajectory. Advanced vision-based methods will measure the 3D shape of the concrete wall in close real time in turbid conditions. To achieve this, the output of each sensor will be enriched with an estimate of perceptual uncertainty, originating from local depth variance (LiDARs) and from stereo block matching and pattern matching quality (cameras). Processed data will be fused through deep learning (recurrent neural networks, gated recurrent units, *etc.*) to provide the system with attention capabilities, attributing to the various input data streams enabling the precise placement of the concrete to the wall.

**Construction robot skills:** The SFR robot will perform the actual spray casting of concrete to the wall. The trajectory planning under disturbances will be provided through an embedded dynamical system (DS) with bifurcation, to switch across single attractor (reaching/retracting) and limit cycles (circular motion) smoothly, using the bifurcation parameters inherited from library of motions constructed in offline skills transferring phase and the initial shotcrete planning model. The planning will consider kinematics and workspace to offer safe interaction with the environment while also closed-loop control will enable concrete extrusion. A hierarchical system will enable the SFR robot to shotcrete a piece of wall up to the required thickness based on open loop policies for the rough completion as well as through vision-enabled closed loop policies to fulfil with concrete up to a desired level based on provided models. Safety during the construction/repair procedure will be ensured (i) by utilizing the appropriate explainable and domain-aware controller during shotcrete operation and (ii) by through human aware navigation.

### Surface finishing phase

**Robot Hardware and Sensors:** The SFR robot will be reused to refine and improve the quality of the shotcreted surface. The robot will be in charge of performing the scrapping step by exploiting specific tools, utilized in the construction domain. During the transition of the construction/repair to the finishing phase, the tools (nozzles *etc.*) might be changed, through custom designed flanges and tool changing approaches.

**Cognitive perception:** A surface finishing visual inspection toolset will evaluate the quality of the shotcreted surface. Information from the nominal BIM/CIM model, the 3D reconstructed one and the monitored surface during the construction/repair procedure will be compared in order to assess the local the quality of the constructed area. Active vision methods through forward kinemics manipulation that guide the wrist-mounted sensor will be applied to explore the surface and assess the depth fidelity through data driven features. These features will measure the concrete granularity, regularity, specularity and other modalities that are indicative of quality.

**Construction robot skills:** The SFR robot will perform the actual surface finishing step to remove concrete from over-spayed areas and to isolate areas which has been under-filled with concrete. The starting point will be the utilization of the construction skills library based on which robot motion primitives will be applied to remove the excessive material. In addition, a shared control scheme that realizes true HRC will be applied, where the workers will be able to intervene and physically interact with the robot to guide it and complete the refining task.

### Digitalization tools

Digitalization tools that will be used through all three shotcrete phases will enable the ambient and easy integration of the robotics cognitive and perception skills with the construction site resources. BIM and CIM models will be enhanced with information from CAD and geographic information system (GIS) system fused with semantic-SLAM information to be visualized in the digital twin platform. A simulation tool that enables interactive 3D simulation of the systems in the construction environment, including kinematic, dynamic and sensor simulation will allow fast robot implementation and testing in simulation environment. The digital twin platform will be a visualization and real time control tool that will effectively communicate in all three phases and represent as-built and as-planned progress discrepancies. A process orchestration method will be responsible to monitor the environment/agents state and propagate the system to the next step based on prompting and trustworthy AI solutions. A dynamic task planning approach will control, activate and monitor the robot perception, cognition and robotic skills. HMI interfaces will enable true collaboration between nozzlemen and robots offering efficient construction outcome as well as increased situation awareness. AR–enabled interaction is also foreseen (projective or optical see-through) to enrich the contextual information during construction/repair/finishing steps.

### Construction sovereignty

In order to distribute the gathered knowledge efficiently and improve the wellbeing of both the construction workforce and construction companies’ specific activities will be deployed, namely, a learning and training environment, a communication platform enhanced with the ability to handle all types of organizational information as well as activities related to relevant standards and social sciences and humanities (SSH) aspects. The learning and training activities are fundamental procedures that will introduce new approaches to the educational capability of construction environments the ability of learning and training will give the opportunity to workforce to assess a dynamically changing base of knowledge for the dynamically changing production requirements, widening in this way the skill profile of employees.

## Targeted application scenarios - Potential use cases

**Figure 5.**
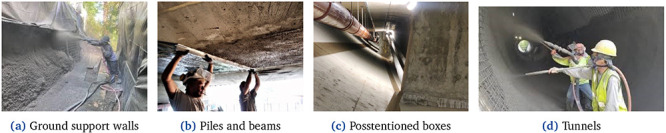
The RoBétArmé use cases. (Images taken by authors at construction sites for the project purposes).

RoBétArmé targets the automation of shotcrete application, which is one of the most demanding tasks in underground mining, tunnel and narrow constructions as well as on buildings complex geometries. The basic steps of dry or wet mix shotcrete are specific in most cases, while differentiations depend on the construction use case. To this end, we juxtapose (in Table 1) the basic steps of the current manual shotcreting with the envisioned RoBétArmé automation steps for each phase. The RoBétArmé will be realized on four selected applications in construction domain that have significant economical and societal impact. In particular within the project the prototypes will be demonstrated in two construction scenarions namely, construction of ground support walls and culverts/ tunnels and two repair scenarios namely, repair of piles and beams and bridges’ post-tensioned boxes. Indicative shtocrete processes in such environments is highlighted in the Figure 5.

**Table 1.** The automation procedure of shotcrete with RoBétArmé project.

Current manual shotcrete procedure	RoBétArmé automated shotcrete procedure
Inspection and preparatory phase	
• Workers manual purge of unstable elements	• Load the Construction/Builidng Information (CIM/BIM) models to digital twin and register IRR robot with GIS measurements
• Placement of the metallic net and manual welding of spacers to avoid deformation under the weight of fresh concrete	• Scan with IRR robot to model the 3D surface
• Placement of bolt-tips and string lines to delimit the area to be shotcreted	• Extract the volumetric area to be shotcreted
• Formwork mounting at the edges/sides if needed	• Populate the digital twin with updated models
• Spray the surface with compressed water	• Simulate, suggest and visualize construction tasks
	• Detect unstable elements and populate the digital twin
	• Detect defects in metallic net and areas to be welded
	• Metal 3D printing to fix deficiencies and spacers in rebar
	• Implant IoT sensors and connect to digital twin
Construction / repair with shotcrete phase	
• Selection and preparation of material mix	• Infer the shotcrete construction plan, including material mix, additives and mix-machine parameters
• Selection of nozzle and hoses types	• Visualization of the plan in digital twin environment, perform corrections by the nozzlemen
• Parameterization of pumpability and projectability in mixture machine and nozzle	• Navigate the SFR robot to the areas of interest and iteratively perform dexterous shotcrete placement, based on learned shotcrete manipulation motions by humans.
• Shotcrete first along the edges to avoid excess material in corners	• Closed-loop vision system of SFR robot controls the thickness
• Manipulate the nozzle with elliptical movement adapted to the distance	• Close to real time comparison of shotcreted surface with Computer Aided Design (CAD)
• Control the thickness by observing the metallic rods or by following the formwork	• Visualize progress and allow control of nozzlemen through teleoperation
Surface finishing phase	
• Workers are scrapping the shotcreted surface to remove excess shotcrete	• Assess shotcreted surface quality with SFR vision system
• Use trowels and mags with long smooth strokes or circular motions	• Surface finishing plan, update the digital twin
• Quickly spray the scrapped surface with a thinner layer without admixture or humidification by spraying water.	• Perform eventually tool changing (scrappers, mags, *etc.*) to the SFR robot
• Manual inspection of the surface quality	• Navigate the SFR robot at the areas of interest and perform the surface finishing with manipulation motions.
	• Engage nozzlemen to guide robot through shared-control
	• Closed-loop SFR vision system assesses the surface roughness
	• Create a high resolution defect (over/under sprayed concrete) map and extract volumetric areas to be covered
	• If needed, change tool and perform the thin shotcrete layer
	• Visualize process in digital twin, IoT analysis and diagnostics

### Construction of ground support walls

The great adaptability of shotcrete makes it extremely desirable for the construction of ground support (retaining) walls, which are rigid walls used for supporting soil. The specific challenges imposed in such construction sites are related to the following. (i) The various illumination conditions (direct sunlight) where the proposed multimodal vision system should operate with robustness to create the surface model, and to perceive the surface quality during shotcrete. (ii) The ground is typically rough with rocks, uneven soil *etc.* that navigation system should compensate, ensuring accurate platform positioning and stability. (iii) Installation of spacers in the preparatory phase should be applied with metal 3D printing IRR robot. (iv) The SFR robot should accommodate hoses of 50mm and it can be capable of performing elliptical movements at the nozzle, retaining 0.6-1.2m distance from the wall, based on the current pipes diameters that used and the current practices in shotcrete performed by nozzlemen.

### Construction of culverts or tunnels

The focus is on construction of tunnels or culverts through shotcrete. The specific challenges imposed in such construction sites are the following. (i) The narrow space in tunnels implies the presence of occlusions, thus the IRR and SFR perception systems should act complementary to create high-precision 3D reconstructions. (ii) The SFR robot should be capable of performing navigation simultaneous with the shotecreting in order to capture the curvature of area to be covered surface. (iii) The excessive rebound (especially in culverts) increases the dust, which should be again addressed by the perception system of SFR robot. (vi) Culvert and tunnels are featureless environments with repetitive patterns, which hinder robot localization, thus IRR, and SFR should rely on fusion of measurements.

### Repair of piles or beams

The shotcrete application is utilized in the repair of piles and beams typically met in residential buildings where restoration and maintenance are required either after building aging or after a crisis event ( *e.g.* fire/earthquake). The specific challenges imposed in such construction sites are related to the following. (i) GIS registration of IRR robot is difficult in absence of GPS signal in indoors environment and the anchoring to the existing survey models should be done with landmarks during SLAM activities. (ii) There is a need for very precise trajectory planning to avoid shotcreting outside the bounded areas, since beams are of particular small diameter. (iii) The SFR robot should accommodate pipes of 32–60 mm diameter, which varies depending on the repair process. (iv) The SFR robot should be capable of performing delicate scrapping (removal of excessive material) and re-shotcreting – *via* tool changing- to accomplice the smooth surface required in such applications.

### Repair of bridges’ posttensioned boxes

The focus is on repair of posttensioned concrete box of bridges with relatively difficult internal access due to spacing limitations. The specific challenges imposed in such construction sites are the following. (i) The illumination conditions are typical low in such environment and the IRR-SFR robots should be capable of operating with artificial illumination. (ii) the IRR and SFR robots should be flexible with high manoeuvrability to address the space limitations and slopping ground. (iii) GPS access is limited and registration anchoring with the environment via versatile SLAM is required, (iv) the vision system should be capable of detecting exposure rebar that needs to be shotcreted. (v) The perception system of SFR robot should compensate the excessive dust occurred from the rebound phenomenon in the narrow area with limited ventilation. (vi) The repair of posttentioned boxes requires a variation of aggregate size which might vary from 4mm (superficial repair) to 8mm (larger volume and depth), which should be accommodated by the mix-machine and pumping system, including additives. (v) The SFR robot should handle hoses of 20mm.

## Conclusions

In this paper the concept of the RoBétArmé project has been presented, where the key challenges that hinder the introduction of digitization and robotics solutions in construction domain, have been firstly presented and the projects overall technological objectives that will tackle this issue have been analytically described. The state of art related to the technologies in the construction domain to be developed have been discussed and the basic architectural elements of the hardware’s and software have been documented. The paper introduced the contribution of RoBétArmé in the automation of construction with shotcrete and presented all the technical advancements that will take place within the project. A state of art analysis for the scientific domains that are involved in the automation of shotcrete has been conducted. The automation procedure has been split into three phases in which different novel robotic mobile manipulators will be developed, *e.g.* IRR and SFR robot. IRR will undertake the modeling and additive manufacturing tasks while SFR will perform the shotcrete and surface finishing. To demonstrate the developed system, four use cases have been selected, in which the introduction of new technologies has significant societal and economical impact, The two use cases are focused in the construction of new structures *i.e.* culverts/tunnels and retaining walls, while the rest two use cases are focused in the repair activities of piles/beams and bridges parts. Last, the provided descriptions in this manuscript aims firstly to raise the attention of the rest of the scientific community regarding the development of new methods to compensate the robot perception and manipulation in challenging construction environments. It also desires to familiarize engineers and stakeholders involved in the construction domain with new technologies to be realized in the close future that will provide important digitilization and automation solutions of current labour-intesive activities.

## Ethics and consent

Ethical approval was not required. Written informed consent was obtained from all participants involved in the included figures.

## Data Availability

No data are associated with this study.
