# Supplementary material for: RoBétArmé Project: Human-robot collaborative construction system for shotcrete digitization and automation through advanced perception, cognition, mobility and additive manufacturing skills
Source: Open Res Eur. 2024 Jan 3;4:4. [Version 1] doi: 10.12688/openreseurope.16601.1 (PMC10879757; doi:10.12688/openreseurope.16601.1)
Supplement: Peer review history

# Reviewer report: Reviewer response for version 1

**DOI:** 10.21956/openreseurope.17922.r37423

**Published:** 2024-02-20

**Authors:** Andrea Orlandini

**Affiliations:** 1 Consiglio Nazionale delle Ricerche (CNR), Rome, Italy

- This manuscript provides an interesting overview about the RoBetArme project envisaging the deployment of advanced technologies to foster the development of advanced additive manufacturing solutions in building construction.
- As it is, the open letter identifies a series of relevant challenges to be addressed related to the specific domain of shotcrete building. The work discusses the most common limitations pointing out some possible solutions.
- The RoBetArme approach is presented and discussed considering advanced robot hardware and sensors coupled with new cognitive perception features. These enable the definition and deployment of enhanced robot skills to be deployed in different construction phases (i.e., design, inspection/preparation, construction/repair and surface finishing.
- The description of four interesting scenarios ends the letter showing potential applications of RoBetArme solutions.
- The manuscript seems interesting but, as far as I can say, it is also quite generic in describing the envisaged methodologies and technologies. The letter does not provide a clear view of how the consortium will pursue the claimed objectives and, as a consequence, it is hard to evaluate the quality of its possible results. Also, the letter mentions some other projects but a deeper analysis of the current state of the art should be provided to better show the advantages of the proposed solution.
- My general feeling is that the paper should provide some more details about the envisage work plan in particular describing which are the considered technologies/solutions over which develop the RoBetArme architecture. 

Where applicable, are recommendations and next steps explained clearly for others to follow? (Please consider whether others in the research community would be able to implement guidelines or recommendations and/or constructively engage in the debate)

Partly

Does the article adequately reference differing views and opinions?

Partly

Are all factual statements correct, and are statements and arguments made adequately supported by citations?

No

Is the rationale for the Open Letter provided in sufficient detail? (Please consider whether existing challenges in the field are outlined clearly and whether the purpose of the letter is explained)

No

Is the Open Letter written in accessible language? (Please consider whether all subject-specific terms, concepts and abbreviations are explained)

Yes

Reviewer Expertise:

autonomous robots

I confirm that I have read this submission and believe that I have an appropriate level of expertise to confirm that it is of an acceptable scientific standard, however I have significant reservations, as outlined above.

# Reviewer report: Reviewer response for version 1

**DOI:** 10.21956/openreseurope.17922.r37429

**Published:** 2024-01-29

**Authors:** Dimitrios Kanoulas

**Affiliations:** 1 University College London, London, England, UK

The open letter, which is based on the RoBétArmé Project, gives a high level view of the project, mainly in general terms. There are few issues to fulfil the need of a good reference in the research community:

 1. Ethical aspect of the project ("rising cost of skills workers"): I think the whole intro, but also later in the text is in the wrong direction. Replacing humans with robots because it is less costly is just the wrong reason to do research in the area, while protecting humans from breathing problems in hazardous environments should be the main reason that drives such a research.

 2. Are the barriers mentioned in the second paragraph handled (all or in part) in the project? It seems a bit challenging, and maybe lowering a bit the bar is needed.

 3. There are some typos such as: "3. (To control the ", "RoBétArmémethodology"

 4. As above, there are some overshooting goals: i) *high-precision and real-time infrastructure perception system: *is this possible? What will be the novel approach compared to what has been tried in the past, especially with cheap sensors? those sensors that are mentioned (LiDAR, RGB) will probably bring uncertain data. ii) where dexterity in manipulation is needed in the task? iii) how Co2 emissions will be decreased compared to the way a worker is doing the job? iv) How spraying will be simulated? This is a challenging topic.

 In general the research details in the methodology are missing; there are just general concepts of robotics. There needs to be something more particular and ground breaking described.

 The rest of the draft looks OK.

Where applicable, are recommendations and next steps explained clearly for others to follow? (Please consider whether others in the research community would be able to implement guidelines or recommendations and/or constructively engage in the debate)

Partly

Does the article adequately reference differing views and opinions?

No

Are all factual statements correct, and are statements and arguments made adequately supported by citations?

Yes

Is the rationale for the Open Letter provided in sufficient detail? (Please consider whether existing challenges in the field are outlined clearly and whether the purpose of the letter is explained)

Partly

Is the Open Letter written in accessible language? (Please consider whether all subject-specific terms, concepts and abbreviations are explained)

Yes

Reviewer Expertise:

Cognitive robotics

I confirm that I have read this submission and believe that I have an appropriate level of expertise to confirm that it is of an acceptable scientific standard, however I have significant reservations, as outlined above.

# Reviewer report: Reviewer response for version 1

**DOI:** 10.21956/openreseurope.17922.r37425

**Published:** 2024-01-25

**Authors:** Konstantinos Tsintotas

**Affiliations:** 1 Democritus University of Thrace, Xanthi, Greece

The RoBétArmé project unfolds as an indispensable and groundbreaking 

 venture, strategically positioned to surmount formidable challenges in integrating digitization and robotics within the construction domain. With meticulous precision, the paper elucidates the project's pivotal role by clearly outlining its ambitious technological objectives. A hallmark of scholarly rigor is observed in the comprehensive analysis of cutting-edge technologies in the construction landscape. At the same time, the intricate architectural components of both hardware and software underscore the project's commitment to exacting detail and innovation.

 Central to RoBétArmé's significance is its visionary approach to automating construction processes, specifically in shotcrete applications. The study introduces a sophisticated tri-phasic strategy featuring the development of groundbreaking robotic mobile manipulators, most notably the Integrated Robotic Rover (IRR) and the Surface Finishing Robot (SFR). The former is designated for modeling and additive manufacturing tasks, while the latter specializes in shotcrete application and surface finishing, showcasing a nuanced understanding of the multifaceted challenges inherent in construction automation.

 Furthermore, the paper strategically accentuates RoBétArmé's societal and economic impact by meticulously selecting four impactful use cases. These span from the construction of culverts/tunnels and retaining walls to the intricate repair activities of piles/beams and bridge components, effectively illustrating the project's real-world applicability and transformative potential. The work not only aspires to captivate the scientific community's attention but also serves as an informative beacon for engineers and stakeholders in the construction domain. By introducing forthcoming technologies to spearhead crucial digitalization and automation solutions, RoBétArmé emerges as a visionary force poised to revolutionize labor-intensive construction activities. The project is a testament to detailed planning, innovative prowess, and a resolute commitment to advancing the construction industry into an era of unprecedented efficiency and technological sophistication.

Where applicable, are recommendations and next steps explained clearly for others to follow? (Please consider whether others in the research community would be able to implement guidelines or recommendations and/or constructively engage in the debate)

Yes

Does the article adequately reference differing views and opinions?

Yes

Are all factual statements correct, and are statements and arguments made adequately supported by citations?

Yes

Is the rationale for the Open Letter provided in sufficient detail? (Please consider whether existing challenges in the field are outlined clearly and whether the purpose of the letter is explained)

Yes

Is the Open Letter written in accessible language? (Please consider whether all subject-specific terms, concepts and abbreviations are explained)

Yes

Reviewer Expertise:

Robotics, Computer vision, Machine learning

I confirm that I have read this submission and believe that I have an appropriate level of expertise to confirm that it is of an acceptable scientific standard.
